# Sleep Disturbances in COVID-19–Positive Healthcare Professionals: Prevalence and Risk Factors

**DOI:** 10.1192/j.eurpsy.2026.11024

**Published:** 2026-07-31

**Authors:** A. Aloui, I. Kacem, A. Ghenim, A. chouchane, M. Bouhoula, M. Makhloufi, H. Kalboussi, O. El Maalel, S. Chatti

**Affiliations:** Department of Occupational Medicine, Farhat Hached University Hospital, Sousse, Tunisia

## Abstract

**Introduction:**

In the context of the COVID-19 crisis, healthcare professionals were particularly exposed to the risk of infection due to the large number of patients they received. This disease involves not only physical symptoms but also psychological symptoms such as insomnia and depression.

**Objectives:**

To determine the prevalence and associated factors of insomnia among healthcare workers affected by COVID-19.

**Methods:**

An analytical cross-sectional study was conducted among healthcare professionals at Farhat Hached University Hospital who were diagnosed with COVID-19 and followed in the Occupational Medicine Department over a six-month period. Data were collected using a questionnaire covering socio-professional characteristics and medical data. Insomnia was assessed using the Insomnia Severity Index (ISI).

**Results:**

A total of 477 cases were included, representing a response rate of 85.9%. The mean age of the study population was 39.95 years, with a predominance of females (78.2%). The professional categories most affected by COVID-19 were nurses (32.1%) and senior technicians (19.9%). According to the ISI score, 43.8% of participants suffered from insomnia. The proportion of women was significantly higher among subjects with insomnia (86.2% vs. 13.8%; p=0.021). Multivariate analysis identified the duration of confinement (OR=1.12, 95% CI \[1.08–1.16]), withdrawal from colleagues upon returning to work (OR=3.5, 95% CI \[2.1–5.8]; p<0.01), and residual symptoms (OR=3.1, 95% CI \[1.9–5.2]; p<0.01) as factors associated with insomnia.

**Conclusions:**

The COVID-19 pandemic disrupted daily life in multiple ways. This study revealed that a significant number of healthcare workers suffered from insomnia. These findings underscore the need to strengthen preventive measures and provide psychological support to healthcare professionals, particularly those who have contracted the disease.

**Disclosure of Interest:**

None Declared

